# Inferior ST-elevation myocardial infarction due to a thrombosed sinus of Valsalva aneurysm

**DOI:** 10.1093/ehjcr/ytaf042

**Published:** 2025-01-27

**Authors:** Taek Jeong, Timothy G Scully, James Theuerle, Ali Al-Fiadh

**Affiliations:** Department of Cardiology, Austin Health, 145 Studley Road, Heidelberg, Victoria 3084, Australia; Department of Cardiology, Austin Health, 145 Studley Road, Heidelberg, Victoria 3084, Australia; Department of Cardiology, Austin Health, 145 Studley Road, Heidelberg, Victoria 3084, Australia; Department of Cardiology, Austin Health, 145 Studley Road, Heidelberg, Victoria 3084, Australia

A 77-year-old male presented with chest pain and an electrocardiogram showing an inferior ST elevation myocardial infarction (see Supplementary material online, *Figure S1*). During coronary angiography, pooling of contrast adjacent to the right coronary cusp was detected with no flow down the right coronary artery (RCA), indicative of dissection or aneurysm (*Figure 1A*, Supplementary material online, *Video S1*). Urgent CT aortogram identified a 7.6 cm outpouching from the right aortic sinus, representing a sinus of Valsalva aneurysm (SOVA). Mixed density within the aneurysm suggested acute thrombus formation, with migration of the thrombus into the RCA as the cause of the patient’s initial presentation (*Figure 1B–F*).

**Figure 1:**
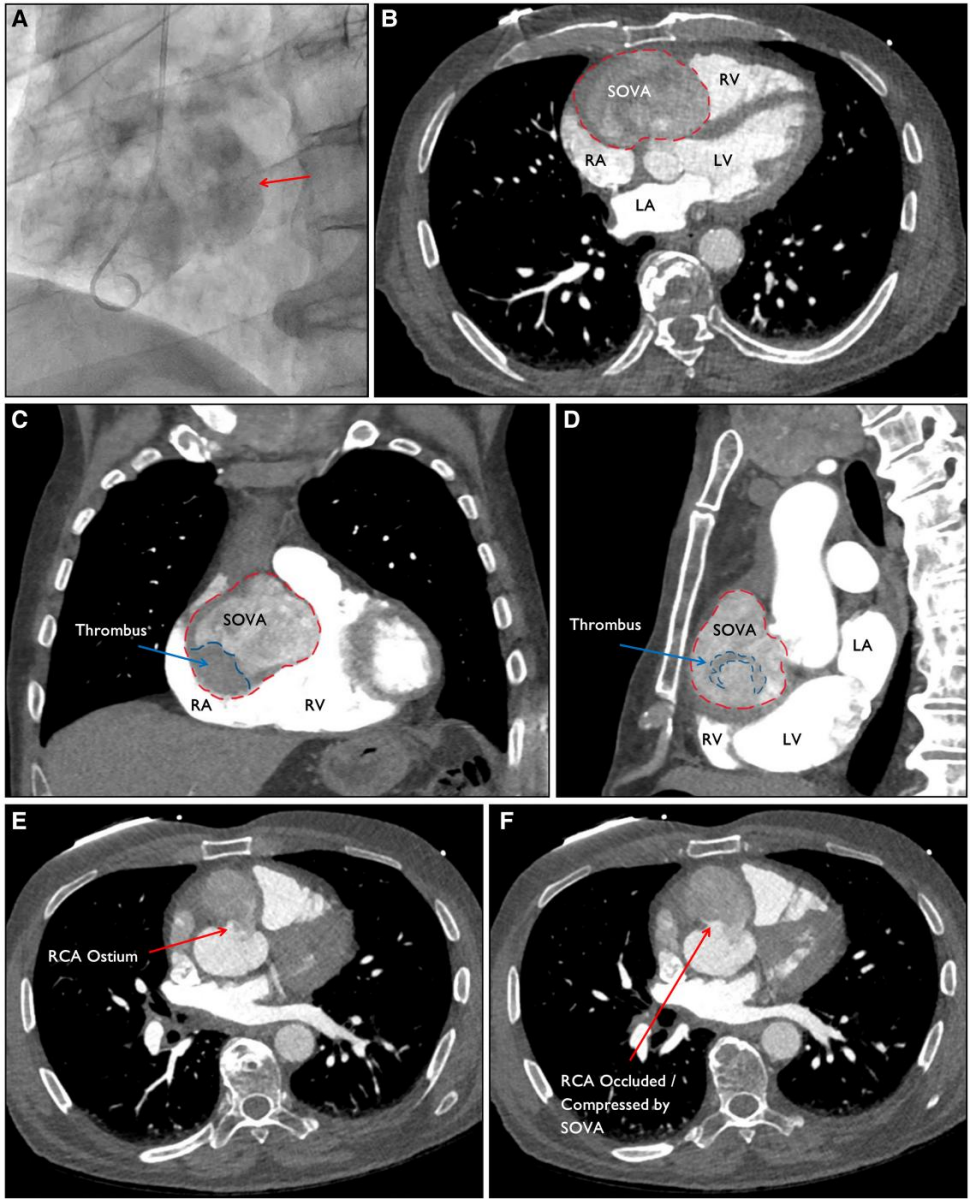
(*A*) Coronary angiogram demonstrated pooling of contrast adjacent to the right coronary cusp (arrow), indicating a dissection/aneurysm. (*B–D*) Computed tomogram aortogram showed a 50 × 67 × 76 mm large aneurysmal dilatation from the right aortic sinus, representing a right sinus of Valsalva aneurysm (dotted red, outer line). Heterogenous contrast mixing within the structure represents probable thrombi (dotted blue, inner line). (*E* and *F*) Transverse slices of the CT chest demonstrating the presence of the right coronary artery ostium, with its eventual occlusion due to probable thrombus or compression by the aneurysm (arrows).

A bedside transthoracic echocardiogram showed compression of the right atrium (RA) and right ventricle (RV) with severe dilatation and reduced function, likely indicating a chronic aneurysm. The patient was deemed an unsuitable surgical candidate due to his severe RV dysfunction and concerns of cognitive impairment. He was commenced on a heparin infusion to reduce thrombus burden within the SOVA, however, died within 24 h of presentation likely due to spontaneous rupture of the aneurysm.

Sinus of Valsalva aneurysm is defined as an abnormal dilatation of the aortic root between the aortic valve annulus and sinotubular junction, caused by weakening of the elastic lamina at the junction of the aortic media and the annulus fibrosis.^1^ It is a rare phenomenon, occurring in ∼0.14% of the population and can be congenital (e.g. connective tissue disorders), or acquired (e.g. syphilis and bacterial endocarditis).^2^

A critical complication of SOVA is rupture, which typically occurs in a trimodal distribution at infancy, between 20 and 40 years of age, and late adulthood. Right coronary SOVAs generally rupture into the RV outflow tract, noncoronary SOVAs into the RA, and left coronary SOVAs into the left atrium or left ventricle. Rupture into the pericardial space may occur from any sinus and is generally fatal.

Traditionally, surgical repair has been the standard treatment for both ruptured and unruptured SOVAs, with 10-year survival rates of ∼90%.^2^ However, emerging evidence indicates transcatheter closure (TCC) using patent ductus arteriosus or Amplatzer occluders is a viable alternative, particularly for high-risk surgical candidates. Whilst patient selection criteria for TCC remains a field of uncertainty, this approach should only be considered in the absence of concomitant heart defects, arrhythmias, or outflow tract obstructions.^3^ Further clinical trials or large-scale studies would be beneficial to establish clear guidelines for indications towards TCC.

## Supplementary Material

ytaf042_Supplementary_Data

## Data Availability

All data are incorporated into the article and its online Supplementary material.

## Notes

**Consent:** Consent for publication was obtained in line with the COPE best practice guidelines.

**Funding:** None declared.
