# Supplementary figures and images for: Inferior ST-elevation myocardial infarction due to a thrombosed sinus of Valsalva aneurysm
Source: Eur Heart J Case Rep. 2025 Jan 27;9(2):ytaf042. doi: 10.1093/ehjcr/ytaf042 (PMC11799936; doi:10.1093/ehjcr/ytaf042)

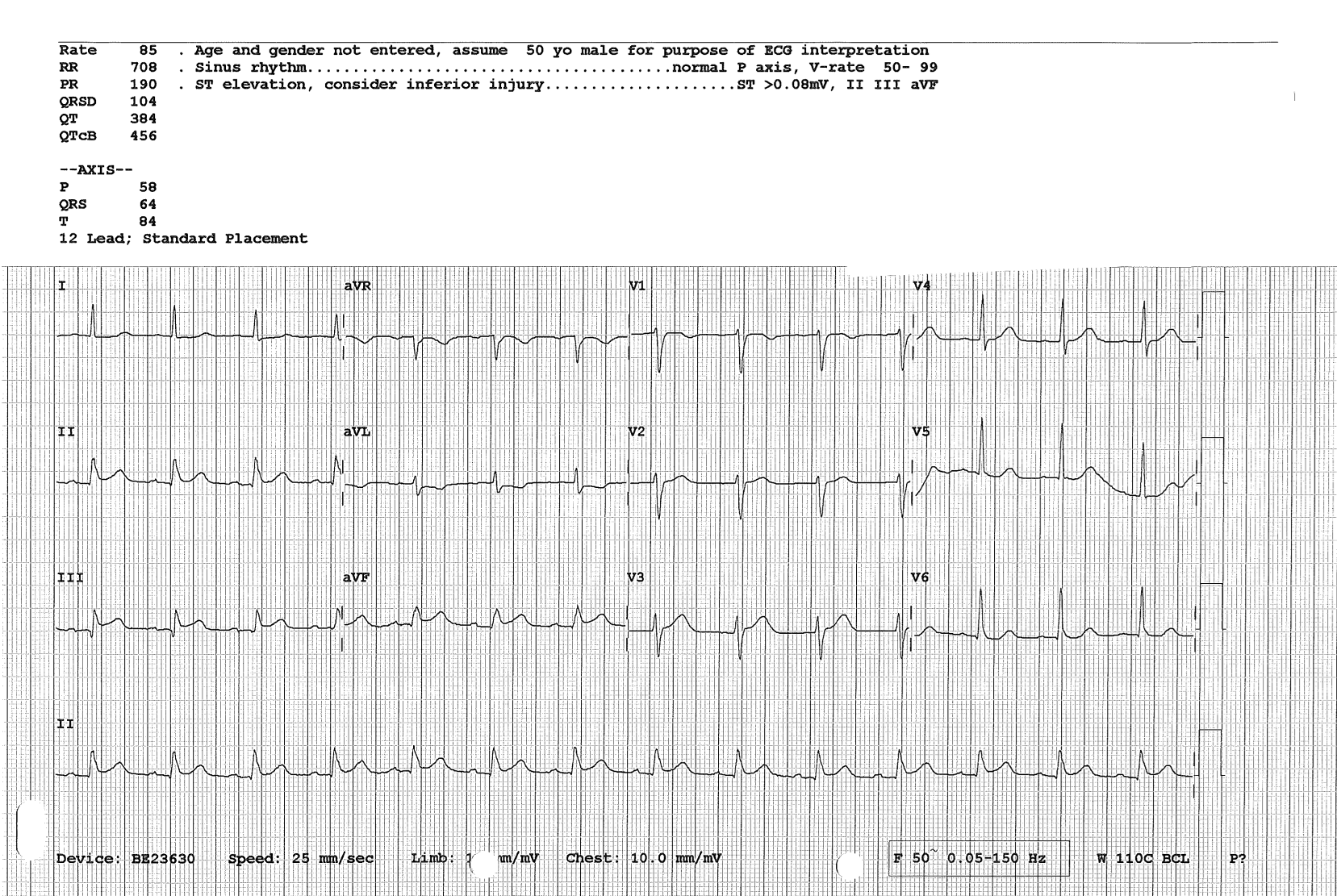

Supplement: ytaf042_Supplementary_Data [file ytaf042_supplementary_data.zip › ECG.png]
